# Fanconi Syndrome Accompanied by Renal Function Decline with Tenofovir Disoproxil Fumarate: A Prospective, Case-Control Study of Predictors and Resolution in HIV-Infected Patients

**DOI:** 10.1371/journal.pone.0092717

**Published:** 2014-03-20

**Authors:** Samir K. Gupta, Albert M. Anderson, Ramin Ebrahimi, Todd Fralich, Hiba Graham, Valeska Scharen-Guivel, John F. Flaherty, Claude Fortin, Robert C. Kalayjian, Anita Rachlis, Christina M. Wyatt

**Affiliations:** 1 Division of Infectious Diseases, Indiana University School of Medicine, Indianapolis, Indiana, United States of America; 2 Division of Infectious Diseases, Emory University School of Medicine, Atlanta, Georgia, United States of America; 3 Gilead Sciences, Inc., Foster City, California, United States of America; 4 Hôpital Notre Dame of Montreal University Medical Center, Montreal, Canada; 5 Division of Infectious Diseases, Metrohealth Medical Center, Cleveland, Ohio, United States of America; 6 Sunnybrook Health Sciences Centre, University of Toronto, Toronto, Canada; 7 Division of Nephrology, Icahn School of Medicine at Mount Sinai, New York, New York, United States of America; University of São Paulo School of Medicine, Brazil

## Abstract

**Objective:**

The predictors of Fanconi syndrome (FS) accompanied by renal function decline with use of the antiretroviral tenofovir disoproxil fumarate (TDF) have not been assessed. In addition, the natural history of renal recovery from FS after TDF discontinuation is not well-described.

**Design:**

We prospectively enrolled HIV-infected patients receiving TDF with newly identified FS (defined as at least two markers of proximal tubulopathy and either a >25% decline in creatinine clearance (CrCl) from pre-TDF values or a CrCl <60 mL/min in those without a known pre-TDF CrCl) in a multicenter observational study. These case participants were matched 1∶2 to controls; characteristics between the two groups were compared. Case participants with known pre-TDF CrCl values were then followed over 48 weeks to assess renal recovery.

**Results:**

Nineteen cases and 37 controls were enrolled. In multivariable analysis, previous or concurrent use of lopinavir/ritonavir [OR 16.37, 95% CI (2.28, 117.68); P = 0.006] and reduced creatinine clearance prior to initiation of TDF [OR 1.44 for every 5 mL/min reduction, 95% CI (1.09, 1.92); P = 0.012; OR 19.77 for pre-TDF CrCl lower than 83 mL/min, 95% CI (2.24, 174.67); P = 0.007] were significantly associated with FS. Of the 14 cases followed for resolution, 7 (50%) achieved at least partial resolution (defined as recovering CrCl >70% of pre-TDF values) although most participants had full normalization of proximal tubulopathy markers within two months of TDF discontinuation.

**Conclusions:**

FS, defined by specific CrCl decreases and markers of tubulopathy, is more likely in those who have received or are currently receiving concomitant lopinavir/ritonavir or who had lower CrCl prior to TDF initiation. Half of those with protocol-defined FS had CrCl recover to near pre-TDF values during the first year after TDF discontinuation.

## Introduction

The use of tenofovir disoproxil fumarate (TDF) is recommended as a component of all first-line treatment regimens in antiretroviral (ART)-naïve, HIV-infected patients due to its high virologic efficacy and good tolerability [1], [2]. However, with the widespread use of TDF, reports of nephrotoxicity, including renal proximal tubulopathy (or Fanconi syndrome), have been published [3]. However, the overall incidence of Fanconi syndrome (FS), especially when accompanied by clinically relevant reductions in renal function, remains low [4], [5]. This suggests there may be specific predisposing factors that put certain patients at risk for more severe forms of TDF-related renal toxicity. Suggested risk factors have included use of concomitant protease inhibitors [6] or didanosine [3], genetic polymorphisms of renal transporters of tenofovir [7], [8], lower weight [9], and greater circulating tenofovir levels [10]. A comprehensive assessment of potential risk factors would be clinically useful to identify those patients for whom FS with actual renal function decline is more likely to occur. In addition, it would be valuable to describe the time to resolution of this type of FS in order to provide clinicians and patients with a better understanding of the natural time course of renal improvement, if any, once TDF is withdrawn, especially as recent reports suggest that full resolution is not universal [11], [12].

Several previous investigations have attempted to answer these important questions. However, these studies were limited in that the definitions of FS were not standardized. Some only used reductions in renal function without corroborative markers of proximal tubulopathy (e.g. proteinuria, normoglycemic glycosuria, hypophosphatemia) that would have increased the likelihood that the nephrotoxicity was truly attributable to TDF [13], [14]. Others used definitions of FS based only on proximal tubulopathy markers using intensive laboratory investigations, many of which are not routinely obtained in practice, without accompanying reductions in renal function [15]. In addition, these studies were necessarily biased towards misclassification as they used only a single set of laboratory findings to identify FS and, thus, could not ensure that the abnormalities identified were persistent [15], [16]. Finally, these studies did not uniformly assess for resolution of both renal function and proximal tubulopathy abnormalities at pre-specified time points or over a standard duration of time after TDF discontinuation [11].

Thus, we present here the results of a prospective, intensive, multicenter, case-control study to determine factors predictive of confirmed TDF-associated FS defined using a standardized definition from commonly obtained clinical laboratories of renal proximal tubulopathy and accompanied by clinically relevant renal function decline. We then systemically assessed the times to resolution of FS (for both the proximal tubulopathy abnormalities and the reduced renal function) to levels documented prior to TDF initiation.

## Methods

### Ethics Statement

All study participants provided written, informed consent. The protocol was approved by the local regulatory body at each participating site (see Table S1 for a complete list of the regulatory bodies which approved the study).

### Study design and population

This phase 4 study (ClinicalTrials.gov NCT00499187) was sponsored by Gilead Sciences, Inc. The academic and industry investigators jointly contributed to the study's design, implementation, and interpretation of results. The manuscript was drafted by S.K.G. with input from all authors. The decision to publish this manuscript was initiated by the academic authors with acknowledgment by the Gilead Sciences authors.

This was a multicenter, case-control (1∶2) study with prospectively enrolled study participants. Fanconi syndrome (FS) cases included study participants receiving TDF who fulfilled the protocol-defined criteria. This definition required the case participant to have had a reduction in creatinine clearance (CrCl), estimated using the Cockcroft-Gault equation [17], of at least 25% from a CrCl estimate obtained within 3 months prior to TDF initiation or a CrCl <60 mL/min if pre-TDF CrCl data were not available. The use of CrCl was chosen as the renal function estimate for this study due its preferred use for dose modification requirements and in accordance with the approved labels for TDF-containing products. In addition, the case participants were required to have at least two of the following laboratory markers of renal proximal tubulopathy: new onset or worsening of pre-existing proteinuria of at least ≥1+ on urinary dipstick; new onset or worsening of pre-existing glycosuria of at least ≥1+ on urinary dipstick with concomitant serum glucose <200 mg/dL; serum potassium <3.0 mEq/L; serum bicarbonate <19 mEq/L in those with a CrCl ≥25 mL/min, and serum phosphorus <2.0 mg/dL. The restrictions of having a concomitant serum glucose <200 mg/dL with glycosuria and of having a CrCl ≥25 mL/min with low serum bicarbonate were amendments to the protocol after early review by the external review committee. These markers of proximal tubulopathy were specifically chosen as they were used in the standard definition of renal proximal tubulopathy in the initial therapeutic and safety trials of tenofovir [18] and because they are easily and routinely obtainable in routine clinical practice. Confirmation of both the reduction in CrCl and the presence of these proximal tubulopathy markers were required within 21 days of the initial laboratory test.

An external review committee (ERC) of three independent experts in the fields of HIV-related renal diseases was included to adjudicate the enrolled cases to determine if the case definition was definitely met without question and to evaluate if full resolution of protocol-defined FS was achieved.

Two control participants were matched to each case participant if they themselves did not fulfill the case definition criteria for FS. Matching criteria included age (≤40 years vs. >40 years), having received TDF for similar duration of time (2–12 months longer than the case participant for cases having <3 years of TDF exposure; ≥3 years for cases with ≥3 years of TDF exposure), and being cared for at the same site as the recruited case participant. Control subjects were not matched on gender, race, and type of antiretroviral regimen as these were considered potential risk factors for the analysis. The control participants completed one study visit to review medical records and collect laboratory data.

The decision to continue or to discontinue TDF after enrollment was made by the participants' HIV caregivers. After the enrollment visit, the case participants for whom a pre-TDF CrCl was known were then followed for resolution of their FS abnormalities. Subjects who continued TDF were followed every 4 weeks for 12 weeks or until resolution was achieved, whereas those who discontinued TDF were followed for up to 48 weeks or until resolution was achieved, whichever occurred first. Full resolution was defined as achieving a CrCl >90% of pre-TDF values. Partial resolution was defined as achieving pre-TDF CrCl values 70–90% of pre-TDF values whereas non-resolution was defined as achieving <70% of pre-TDF CrCl values within 48 weeks of follow-up. The final follow-up CrCl value may have been based on just one measurement. Proximal tubulopathy markers were also assessed at each study visit for resolution.

All clinical laboratories were assessed centrally (Covance Central Laboratory Services, Inc.; Indianapolis, IN) to confirm fulfillment of the case definition and during the resolution phase of the study.

### Statistical analysis

Descriptive statistics were used to characterize the profiles of cases and controls included in the study. The data for the following variables were reviewed for risk of Fanconi syndrome: age, sex, race, history of diabetes or hypertension, co-infection with hepatitis B or hepatitis C, time since HIV diagnosis, time on any antiretroviral therapy, CD4 count, pre-TDF CrCl, weight, specific laboratory assessments other than creatinine (ALT, AST, albumin, bilirubin, white blood cells, platelets), use of any protease inhibitor (prior, current, and/or ever), use of lopinavir/ritonavir specifically (prior, current, and/or ever), use of atazanavir specifically (prior, current, and/or ever), and duration of TDF receipt.

Logistic regression models were utilized to identify potential predictive factors of being a case participant vs being a control participant. The model building was based on initial review of the descriptive statistics followed by a series of univariable and multivariate analysis steps. This sequential approach to modeling was planned since we expected missing results for different variables across different subjects; as such, we wished to minimize information loss from this small study. The selections were based on an entry significance level of 0.3 and were retained in the model at a significance level of 0.2 for exploratory purposes. With this approach, the following characteristics were selected for statistical modeling: pre-TDF CrCl, concurrent use of any HIV protease inhibitor with TDF, past or concurrent use specifically of atazanavir, and past or concurrent use specifically of lopinavir/ritonavir.

## Results

Fourteen clinical sites were initially involved in this study. Despite intensive monitoring for FS cases, only 19 cases across 9 sites (7 in the U.S. and 2 in Canada) met the definition of confirmed FS and were enrolled as cases between December 2007 and March 2010. A total of 37 participants were enrolled as controls (one case had only one matching control). Of these 19 cases, 14 had pre-TDF CrCl data available and, thus, were eligible to be followed for resolution. The ERC determined that 13 of the 19 cases were unequivocally TDF-related FS. The ERC could not determine with certainty that 6 cases were attributable to TDF for the following reasons: lack of pre-TDF urine analysis in three cases; possible contribution of hyperglycemia (serum glucose >300 mg/dL) to glycosuria in one case; presence of likely acute tubular necrosis in one case; and low bicarbonate in the setting of a CrCl <25 mL/min at presentation in one case. Given the overall low enrollment, the analyses were based on all 19 enrolled cases as each did meet the protocol-defined entry criteria.

Table 1 shows the Fanconi syndrome criteria at presentation for the cases. Of the 19 cases, 14 met the requirement of having a >25% decline in CrCl from pre-TDF values; the other 5 had an enrollment CrCl values <60 mL/min with lack of known pre-TDF values. The median (interquartile range, IQR) pre-TDF and enrollment values for CrCl for the control participants were 107 (88–122) mL/min and 109 (87–121) mL/min, respectively. Proteinuria was the proximal tubulopathy marker most commonly detected, followed by glycosuria, low serum phosphorus, low serum bicarbonate, and low serum potassium.

**Table 1:** Fanconi Syndrome Criteria of Cases at Presentation.

Criteria	All Cases (n = 19)	ERC Cases (n = 13)
Pre-TDF CrCl (mL/min), median (IQR)	80 (65–106)^a^	101 (78–107)
CrCl at FS presentation (mL/min), median (IQR)	42 (19–58)	47 (40–62)
Proteinuria, n (%)	15 (79)	11 (85)
Glycosuria, n (%)	7 (37)	6 (46)
Serum potassium <3.0 mEq/L	2 (11)	1 (8)
Serum bicarbonate <19 mEq/L	2 (11)	2 (15)
Serum phosphorus <2.0 mg/dL	5 (26)	5 (39)

Abbreviations: IQR, interquartile range; TDF, tenofovir disoproxil fumarate; CrCl, creatinine clearance; FS, Fanconi syndrome; ERC, external review committee.

a n = 14 as 5 pre-TDF CrCl values were not available.

The clinical characteristics of the cases and controls are shown in Table 2. Race, age, sex, history of hypertension or diabetes, and hepatitis B or C co-infections were similar between the FS cases and controls. FS cases had longer known duration of HIV infection and had enrollment HIV-1 RNA levels <50 copies/mL less frequently compared to controls. Median pre-TDF CrCl values were lower in the FS cases [80 (IQR, 65–106)] mL/min compared to controls [107 (IQR, 88–122)] mL/min. Duration of current TDF use was somewhat longer in the controls compared to the FS cases. Protease inhibitor-based regimens were more frequent in the cases than controls, especially at the time of FS presentation. Lopinavir/ritonavir and atazanavir use were similar amongst the cases, but both were prescribed more frequently in cases than in controls. There were no appreciable differences between cases and controls in previous or current use of didanosine, indinavir, angiotensin-converting enzyme inhibitors or angiotensin receptor blockers. However, prior or current use of fenofibrate, gastric acid suppressing agents, and non-steroidal anti-inflammatory drug use was more frequent in cases compared to controls.

**Table 2:** Clinical Characteristics of the Fanconi Syndrome Cases and Controls.

Characteristic	All Cases (n = 19)	ERC Cases (n = 13)	Controls (n = 37)
Male sex, n (%)	14 (74)	10 (77)	33 (89)
Age at enrollment (years), median (IQR)	54 (41–60)	54 (41–60)	49 (43–54)
Black race, n (%)	7 (37)	3 (23)	14 (38)
Hepatitis B or C co-infection, n (%)	5 (26)	3 (23)	10 (27)
History of hypertension, n (%)	6 (32)	4 (31)	8 (22)
History of diabetes, n (%)	2 (11)	1 (8)	0 (0)
Years since HIV diagnosis at time of enrollment, median (IQR)	14 (8–20)	17 (8–20)	10 (4–18)
HIV-1 RNA level <50 copies/mL at enrollment, n (%)	15 (79)	11 (85)	34 (92)
Pre-TDF CrCl (mL/min), median (IQR)	80 (65–106)^a^	101 (78–107)	107 (88–122)
Duration of current TDF regimen at enrollment (years), median (IQR)	1.2 (0.6–2.1)	1.5 (0.6–3.7)	1.8 (1.3–3.0)
PI-based regimen at any time prior to or at enrollment, n (%)	16 (84)	11 (85)	28 (76)
PI-based regimen at enrollment, n (%)	16 (84)	11 (85)	19 (51)
Lopinavir-based regimen at any time prior to or at enrollment, n (%)	13 (68)	9 (69)	10 (27)
Lopinavir-based regimen at enrollment, n (%)	8 (42)	5 (39)	7 (19)
Atazanavir-based regimen at any time prior to or at enrollment, n (%)	10 (53)	8 (62)	10 (27)
Atazanavir-based regimen at enrollment, n (%)	6 (32)	6 (46)	7 (19)
Didanosine-based regimen at any time prior to enrollment^b^, n (%)	5 (26)	4 (31)	9 (24)
Indinavir-based regimen at any time prior to enrollment^b^, n (%)	4 (21)	3 (23)	7 (19)
Fenofibrate use at any time prior to or at enrollment, n (%)	4 (21)	3 (23)	0 (0)
Angiotensin-converting enzyme or angiotensin receptor blocker use prior to or at enrollment, n (%)	4 (21)	3 (23)	6 (16)
NSAID use at any time prior to or at enrollment, n (%)	4 (21)	4 (31)	3 (8)
Gastric acid suppressing drugs^c^, n (%)	4 (21)	2 (15)	1 (3)

Abbreviations: ERC, external review committee; IQR, interquartile range; TDF, tenofovir disoproxil fumarate; FS, Fanconi syndrome; PI, protease inhibitor; N/A, not applicable; NSAID, non-steroidal anti-inflammatory drug.

a n = 14 as 5 pre-TDF CrCl values were not available.

b Neither didanosine nor indinavir was used in any study participant at enrollment.

c Includes omeprazole, esomeprazole, famotidine, pantoprazole, and sodium bicarbonate.

In our final multivariable model including all 19 case participants, only previous or concurrent use of lopinavir/ritonavir [OR 16.37, 95% CI (2.28, 117.68); P = 0.006] and lower creatinine clearance prior to initiation of TDF [OR 1.44 for every 5 mL/min reduction, 95% CI (1.09, 1.92); P = 0.012] were independently associated with being a case participant compared to being a control. To provide a more clinically useful model, we then categorized pre-TDF CrCl at 83 mL/min, which is the 33^rd^ percentile across all cases and controls. In this simplified model, previous or concurrent use of lopinavir/ritonavir [OR 23.21, 95% CI (2.53, 212.93); P = 0.005] and pre-TDF CrCl lower than 83 mL/min [OR 19.77, 95% CI (2.24, 174.67); P = 0.007] were independently associated with being a case participant compared to being a control.

All 14 FS cases eligible for follow-up eventually had their TDF discontinued by their HIV provider following their enrollment visit (12 had TDF discontinued at or around the enrollment visit, 1 discontinued 8 weeks after enrollment, and 1 discontinued 12 weeks after enrollment). Four participants discontinued study participation prior to achieving either CrCl resolution or 48 week follow-up due to investigator discretion, withdrawal of consent, loss to follow-up, or protocol violation (one subject each). Four (29%) participants achieved complete resolution of CrCl within 48 weeks after discontinuation of TDF, 3 had partial resolution, and 7 had no resolution. Of those who had renal proximal tubulopathy markers assessed during the 48 week follow-up period, the following markers resolved: 9 of 14 with proteinuria, 8 of 8 with glycosuria, 2 of 2 with hypokalemia, 1 of 2 with hypobicarbonatemia, and 4 of 4 with hypophosphatemia. Except for the 5 cases with persistent proteinuria and 1 case with persistent low serum bicarbonate, resolution of renal proximal tubulopathy markers occurred within 8 weeks of discontinuation of TDF.

Table 3 shows the characteristics of the 7 participants with either complete or partial resolution of CrCl after TDF discontinuation vs. the 7 participants without resolution. These two groups were generally similar although several differences were notable. Those with complete or partial resolution were more likely to be free of either hepatitis B or C co-infection, have lower CrCl at time of TDF discontinuation, have less pronounced dipstick proteinuria, have shorter duration of TDF use prior to study enrollment, and more likely to continue ART of any kind after TDF discontinuation.

**Table 3:** Clinical Characteristics of Those Who Had Complete or Partial Resolution vs. No Resolution after Tenofovir Discontinuation.

Characteristic	Complete/Partial Resolution (n = 7)	No Resolution (n = 7)
Male sex, n (%)	5 (71)	4 (57)
Age at enrollment (years), median (IQR)	55 (53–60)	54 (41–61)
Black race, n (%)	2 (29)	2 (29)
Hepatitis B or C co-infection, n (%)	1 (14)	3 (43)
History of hypertension, n (%)	3 (43)	3 (43)
History of diabetes, n (%)	0 (0)	2 (29)
Years since HIV diagnosis at time of enrollment, median (IQR)	15 (9–22)	14 (8–20)
HIV-1 RNA level <50 copies/mL at enrollment, n (%)	6 (86)	4 (57)
CD4 cell count <200/μL, n (%)	1 (14)	2 (29)
CrCl (mL/min) at TDF discontinuation, median (IQR)	39 (21–68)	46 (33–61)
Proteinuria at TDF discontinuation, n (%)		
1+	4 (57)	0 (0)
2+	3 (43)	5 (71)
3+	0 (0)	2 (29)
Duration of current TDF regimen at enrollment (years), median (IQR)	0.8 (0.1–2.3)	1.9 (1.0–6.1)
PI-based regimen at any time prior to or at enrollment, n (%)	7 (100)	6 (86)
PI-based regimen at enrollment, n (%)	7 (100)	6 (86)
Lopinavir-based regimen at any time prior to or at enrollment, n (%)	7 (100)	4 (57)
Lopinavir-based regimen at enrollment, n (%)	3 (43)	4 (57)
Atazanavir-based regimen at any time prior to or at enrollment, n (%)	6 (86)	2 (29)
Atazanavir-based regimen at enrollment, n (%)	3 (43)	1(14)
Taking any ART after TDF discontinuation, n (%)	6 (86)	3(43)
NRTI only regimens, n (%)	4 (57)	1(14)

Abbreviations: CrCl, creatinine clearance; PI, protease inhibitor; ART, antiretroviral therapy.

## Discussion

In this first, prospective, controlled evaluation of TDF-associated FS, we used a standardized definition to assess readily identifiable characteristics of patients who are more likely to develop FS, which we defined as having a reduction in creatinine clearance accompanied by routinely available markers of renal proximal tubulopathy. We found that previous or current use of lopinavir/ritonavir (but not other protease inhibitors) and lower CrCl at TDF initiation were significantly and independently associated with the development of FS. The associations between TDF-related renal disease with use of lopinavir/ritonavir [6] has been observed previously, but the relationship with reduced initial renal function has not been as consistent [19], [20]. Increased systemic tenofovir levels may lead to worsening renal function [21] and may occur from both concomitant PI use, possibly via inhibition of tenofovir renal clearance [22], or from reduced initial renal function [23].

Although black race is the primary factor related to the development of HIV-associated nephropathy [24], neither this study nor others (to our knowledge) have found that race is associated with TDF-related renal toxicity. Sex and concomitant didanosine use were also not associated with FS in this study. Also of note, hepatitis B or C co-infection, diabetes, and hypertension were not found more commonly in those fulfilling the case definition vs controls. These conditions, which are more frequently associated with acute and chronic kidney injury in HIV [25]–[27], may not be predisposing conditions for the development of TDF-related FS, although the small numbers in our study do not provide conclusive evidence. It is not surprising that neither age nor duration of TDF use was associated with FS in our study as these were matching criteria between cases and controls.

Importantly, we found that only half of the case participants achieved either full or partial recovery of renal function, defined as >70% of their pre-TDF CrCl, within one year of TDF discontinuation. However, the majority of the proximal tubulopathy markers did resolve within 8 weeks of stopping TDF, although proteinuria persisted in several participants. These findings are in agreement with other studies that suggest that markers of proximal tubulopathy induced by TDF can improve relatively quickly, i.e. within 1–2 months, but that full renal function recovery, as estimated by CrCl or eGFR, may only occur in nearly half of the patients affected [11], [28]. Interestingly, several factors appeared to be more frequent in those who had full or partial recovery in our study. Similar to results from both Wever et al [11] and Yoshino et al [12], our findings suggest a shorter duration of TDF prior to discontinuation is associated with a greater likelihood of renal recovery. We also observed that lower level dipstick proteinuria, a marker of less severe renal injury, at time of TDF discontinuation was also a favorable indicator for eventual renal function recovery.

Interestingly, although PI use was associated more often with being a case participant, it was not more frequently found in those who did not recover from FS. Specifically, our data did not suggest that use of atazanavir, which has recently been associated with chronic kidney disease [29], was associated with poorer likelihood of renal recovery. Both our finding of greater likelihood of renal recovery in those who had concomitant PI use and in those with lower CrCl at time of TDF discontinuation are in agreement with those from Wever et al [11]. A novel finding in our study was that continuation of some sort of antiretroviral therapy after TDF discontinuation as opposed to withholding treatment altogether may be important in restoring renal function, possibly by limiting the deleterious effects of untreated viremia on the kidney [30].

Several limitations to this study should be noted. Despite the purported frequency of TDF-related renal disease, very few protocol-defined FS cases were identified in our multicenter study network, which led to less power to find differences between cases and controls and to identify factors associated with lack of renal recovery once TDF is discontinued. We believe this was due in part to our protocol definition which required a confirmed reduction in creatinine clearance with evidence of proximal tubulopathy without evidence of an alternative etiology besides TDF use. Despite this strict definition, the external review committee still had difficulty in confirming that all initially included cases truly reflected FS and were exclusively due to TDF, primarily due to a lack of available pre-TDF clinical data. As such, we also acknowledge that we do not know if TDF initiation actually caused the decline in renal function or if other etiologies of renal dysfunction were already in place when TDF was used. It would also been of interest to capture data on the total numbers of patients receiving TDF at the clinical trial sites and the numbers of patients who met our case definition at a single time point but then did not demonstrate confirmed Fanconi syndrome with renal function decline on subsequent testing. A prospective cohort study design with ongoing case ascertainment certainly would have precluded these limitations. However, such a study would have required following thousands of patients for several years, which would have been difficult and expensive to implement, and would not have addressed the inherent inability of observational studies to establish causality. Our follow-up data after TDF discontinuation was prospective and allowed a systematic evaluation of resolution of the FS criteria. This is a major strength of the current study and allows more confident interpretation and external generalizability. We also acknowledge that routinely available measures of urinalysis protein and glucose are not quantitative measures, and that including those with simply an increase in proteinuria or glycosuria could lead to misclassification of some cases. Nonetheless, we felt that it was clinically important to include participants with pre-existing proteinuria and glycosuria with misclassification minimized by the requirement for at least one additional marker of proximal tubulopathy. Finally, our study was conducted in HIV-1-infected patients, so the results are not directly applicable to other populations wherein TDF is used (e.g. pre-exposure prophylaxis for HIV, chronic HBV monoinfection).

In summary, we found that FS was associated with lopinavir/ritonavir use and lower CrCl at time of TDF initiation. Only half of those followed for 48 weeks after TDF discontinuation had either partial or complete resolution of their renal function, although most did have resolution of their proximal tubulopathy markers. Our findings will allow HIV caregivers to understand better those characteristics associated with TDF renal toxicity and the natural history of renal recovery after TDF discontinuation.

## Supporting Information

Table S1 **Regulatory bodies approving this study.**
(DOC)
