# Supplementary material for: Fanconi Syndrome Accompanied by Renal Function Decline with Tenofovir Disoproxil Fumarate: A Prospective, Case-Control Study of Predictors and Resolution in HIV-Infected Patients
Source: PLoS One. 2014 Mar 20;9(3):e92717. doi: 10.1371/journal.pone.0092717 (PMC3961428; doi:10.1371/journal.pone.0092717)
Supplement: Table S1 — Regulatory bodies approving this study. (DOC) [file pone.0092717.s001.doc]

**Table S1. Regulatory bodies approving this study.**

| **Geographic Region/ Country**  **Principal Investigator (Site Number)** | **Regulatory body** |
| --- | --- |
| **North America/ Canada**  Claude Fortin, MD (2885) | Comite d’ethique de la recherché du CHUM, Hopital Saint-Luc  Edifice Cooper,  3981 Boulevard St-Laurent,  Mezzanine 2- Bureau, M-207  Montreal, QC H2W 1Y5 CANADA |
| **North America/ Canada**  Julio Montaner, MD, FRCPC, FCCP (0543) | UBC/Providence Health Care Research Ethics Board  St. Paul’s Hospital  11^th^ Floor  1190 Hornby St.  Vancouver, BC V6Z 2K5 CANADA |
| **North America/ Canada**  Anita Rachlis, MD (0573) | Sunnybrook Health Sciences Centre REB  2075 Bayview Avenue, C8-19  Toronto, Ontario M4N 3M5 CANADA |
| **North America/ United States**  Albert M. L. Anderson, MD (3056) | Emory University Institutional Review Board  1599 Clifton Road  5th Floor East  Atlanta, GA 30322 USA  Mailstop: 1599-001-1AV |
| **North America/ United States**  Mohamed Atta, MD (2077) | Johns Hopkins Medicine Institutional Review Boards  Reed Hall B-130  1620 McElderry St.  Baltimore, MD 21205 USA |
| **North America/ United States**  Indira Brar, MD (1534) | Henry Ford Health Systems Institutional Review Board  2799 West Grand Blvd.  CFP Basement, Room 046  Detroit, MI 48202 USA |
| **North America/ United States**  Joseph Gathe, Jr., MD (0031)  Benjamin Young, MD, PhD (1541) | Aspire IRB  9320 Fuerte Drive, Suite 105  La Mesa, CA 91941 USA |
| **North America/ United States**  Barbara Gripshover, MD (0442) | University Hospitals Case Medical Center  Institutional Review Board  11100 Euclid Ave.  Cleveland, OH 44106 USA |
| **North America/ United States**  Samir Gupta, MD, MS (2146) | Indiana University Institutional Review Board  620 Union Dr., Suite 618  Indianapolis, IN 46202 USA |
| **North America/ United States**  Dushyantha Jayaweera, MD (1692)  Wilbert Jordan, MD, MPH (0804) | Western Institutional Review Board  3535 Seventh Ave., SW  Olympia, WA 98502 USA |
| **North America/ United States**  Robert Kalayjian, MD (1908) | MetroHealth Institutional Review Board  2500 MetroHealth Drive  RABO 103  Cleveland, OH 44109 USA |
| **North America/ United States**  Christina Wyatt, MD (2801) | Mount Sinai School of Medicine Institutional Review Board  “Program for the Protection of Human Subjects”  One Gustave L. Levy Place, Box 1075  New York, NY 10029 USA |
